# Development of validated stability-indicating chromatographic method for the determination of fexofenadine hydrochloride and its related impurities in pharmaceutical tablets

**DOI:** 10.1186/1752-153X-5-76

**Published:** 2011-12-03

**Authors:** Hadir M Maher, Maha A Sultan, Ileana V Olah

**Affiliations:** 1 Department of Pharmaceutical Chemistry, College of Pharmacy, King Saud University, Riyadh 11495, P.O. Box 22452, Saudi Arabia; 2 Department of Pharmaceutical Analytical Chemistry, Faculty of Pharmacy, University of Alexandria, El-Messalah, Alexandria 21521, Egypt

## Abstract

A simple reversed phase high performance liquid chromatographic method with diode array detector (HPLC-DAD) has been developed and subsequently validated for the determination of fexofenadine hydrochloride (FEX) and its related compounds; keto fexofenadine (Impurity A), meta isomer of fexofenadine (Impurity B), methyl ester of fexofenadine (Impurity C) in addition to the methyl ester of ketofexofenadine (Impurity D). The separation was based on the use of a Hypersil BDS C-18 analytical column (250 × 4.6 mm, i.d., 5 μm). The mobile phase consisted of a mixture of phosphate buffer containing 0.1 gm% of 1-octane sulphonic acid sodium salt monohydrate and 1% (*v/v*) of triethylamine, pH 2.7 and methanol (60:40, *v/v*). The separation was carried out at ambient temperature with a flow rate of 1.5 ml/min. Quantitation was achieved with UV detection at 215 nm using lisinopril as internal standard, with linear calibration curves at concentration ranges 0.1-50 μg/ml for FEX and its related compounds. The optimized conditions were used to develop a stability-indicating HPLC-DAD method for the quantitative determination of FEX and its related compounds in tablet dosage forms. The drugs were subjected to oxidation, hydrolysis, photolysis and heat to apply stress conditions. Complete separation was achieved for the parent compounds and all degradation products. The method was validated according to ICH guidelines in terms of accuracy, precision, robustness, limits of detection and quantitation and other aspects of analytical validation.

## Background

**Figure 1:**
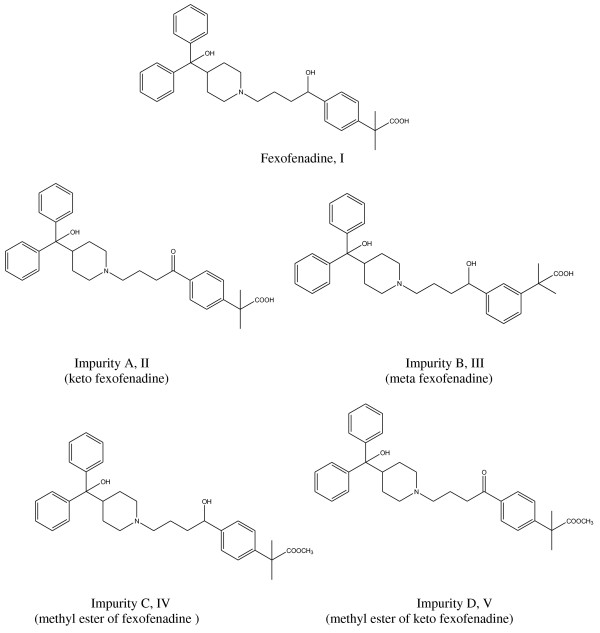
**Structures of fexofenadine and its related compounds**.

Fexofenadine, α, α-dimethyl-4-[1-hydroxy-4-[4-(hydroxydiphenyl-methyl)-1-piperidinyl] butyl]-benzene acetic acid [1] (Figure 1) is the active carboxylic acid analogue of the antihistamine terfenadine. It shares the histamine H1 receptor antagonist and non-sedative properties of the parent compound. This could be attributed to its capability to exist in zwitter-ionic form so it cannot pass through blood-brain barrier and therefore does not cause sedation [2,3]. Fexofenadine is a second generation antihistamine drug useful to available treatments of allergic diseases with a wide margin of safety [4,5]. FEX displays some anti-inflammatory properties and it has also another advantage as it lacks the cardiotoxic side effects (fatal arrhythmia) associated with terfenadine [2,3]. FEX is rapidly absorbed with a long duration of action, making it suitable for once daily administration. Thus, it fulfils the essential and desirable characteristics of an ideal antihistamine, being responsible for the improvement in quality of life of the patients with allergic diseases [4,6].

There are several reports on liquid chromatographic determination of FEX in biological fluids. Among which are those using LC-MS/MS [7], ultraviolet detection [8] and fluorescence detection [9]. Few methods reported the quantitation of FEX in pharmaceutical dosage forms using spectrophotometric methods [10], LC methods with ultraviolet detection [11-13], and capillary electrophoresis [14,15].

It is reported in the BP that four main impurities may be encountered along with FEX (para isomer). These impurities are; impurity A (keto fexofenadine), impurity B (meta-isomer of FEX), impurity C (methyl ester of fexofenadine) and D (methyl ester of keto fexofenadine), Figure 1[16]. The BP describes two separate HPLC methods for the determination of FEX and its four related impurities. The first one utilizes β-cyclodextrin modified silica (silica gel BC for chiral chromatography R1) to test for impurity B while the other is based on an isocratic elution using phenylsilyl silica gel column for the analysis of FEX along with the other three impurities; A, C, D [16]. FEX is also official in the USP [17]. Two HPLC methods have also been described in which one of the methods is used for the separation and determination of related compound-B using an expensive β-cyclodextrin modified silica column (USP L45) and the other method for the determination of both FEX and its related compound A using a phenyl bonded column (USP L11). The other two impurities C and D are not official in the USP [17].

Literature survey revealed a few methods for the quantitative determination of FEX along with its related impurities A and/or B in bulk or pharmaceutical dosage forms by HPLC with UV detection [18,19]. One work [18] describes simultaneous determination of FEX and its two impurities A and B using C8 column as stationary phase and a mobile phase comprising 1% triethylamine phosphate (pH 3.7), acetonitrile and methanol in the ratio 60:20:20 (*v/v/v*). In the other published work [19], the separation between FEX and its related impurity B depends on the use of C18 column. The mobile phase was a mixture of buffer and acetonitrile containing β-cyclodextrin. None of these methods [18,19] deals with impurities C and D.

However, the other impurities C (methyl ester of fexofenadine) and D (methyl ester of keto fexofenadine) may be encountered along with the parent drug FEX depending on the method of preparation (Figure 1). It was reported that FEX was prepared through a process that involves reduction of a compound of formula (II) and isolation of FEX monohydrate in crystalline form. Suitable reducing agents preferably sodium borohydride is used to yield the sodium salt of FEX which is converted to the free base by pH adjustment. Then optionally converting FEX free base to a pharmaceutically accepted salt. A further method for the preparation of FEX comprises a process involving the hydrolysis of a keto ester of formula (V) to the corresponding keto acid of formula (II) and subsequent reduction of the acid as described above [20,21]. This gives the possibility of the existence of impurities A and D, respectively along with the parent FEX. Also, FEX could be produced by a simple hydrolysis of the methyl ester of FEX (a compound of formula IV) giving a chance for the existence of the parent methyl ester, the so called "Impurity C" along with the parent drug.

To our knowledge, no single method was reported for the simultaneous determination of FEX and its related compounds A, B, C and D (Figure 1). The objective of this work was to develop an analytical HPLC procedure, which would serve as reliable and rapid method for the simultaneous determination of FEX and its four related impurities A, B, C and D. This manuscript describes the development and subsequent validation of an isocratic reversed phase HPLC method with diode array detector using C 18 column as stationary phase for the above determination. Quantitation was achieved using lisinopril (LIS) as internal standard. In the proposed HPLC method, the four impurities were well separated from FEX and eluted before 25 min run time. The stability-indicating property of the proposed method was also evaluated.

## Experimental

### Materials and Reagents

Samples of fexofenadine hydrochloride (FEX) and its related compounds A, B, C and D along with the internal standard (lisinopril, LIS) were kindly supplied by Drug control centre, Riyadh, Saudi Arabia. All reagents used were of analytical grade, namely: Methanol (Panreac Co., E.U.), 1-octane sulphonic acid sodium salt, ortho-phosphoric acid and triethylamine (BDH Laboratory Suppliers, Poole, England). The water used was double glass distilled. Phosphate buffer solutions (0.05 M) of different pH values were tried.

Tablets of Telfast^® ^(Safani Aventis, Paris, France), 120 mg of active drug FEX, batch number: 0TOO757, were obtained through local pharmacy.

### Instrumentation and chromatographic conditions

The chromatographic system, Waters (Milford, MA 01757, USA) consisted of Waters 1525 Binary HPLC Pump, Waters 2707 Autosampler fitted with a 20 μl sample loop and Waters Diode array detector with multiple wavelength selector. The LC system is equipped with a data handling system comprised of a Dell personal computer and empower 2 software. HPLC separations were performed on a Hypersil BDS stainless-steel C-18 analytical column (250 × 4.6 mm, i.d.) packed with 5 μm diameter particles. The mobile phase was a mixture of an aqueous phase and methanol in a ratio of 60: 40 (*v/v*). The aqueous phase consisted of 0.05 M phosphate buffer containing 0.1 gm% of 1-octane sulphonic acid sodium salt monohydrate and 1% (*v/v*) of triethylamine. The pH of the aqueous phase was adjusted to pH 2.7 with orthophosphoric acid solution (10%). The mobile phase was filtered through a Millipore membrane filter (0.2 μm) from Nihon, Millipore (Yonezawa, Japan), and was degassed before use. The flow rate was 1.5 ml/min. The detection wavelength was set at 215 nm and at ambient temperature (25°C). The injection volume was 20 μl. The quantitation was performed using lisinopril as internal standard. The area ratio was calculated relative to the internal standard (LIS).

### Standard solutions and Calibration Graphs

Stock solutions (50 mg%) of FEX and its related compounds were prepared in methanol. These solutions were further diluted with the mobile phase to obtain working standard solutions of suitable concentrations (0.1-50 μg/ml for both FEX and the four related substances). The concentration of the internal standard was maintained at 30 μg/ml in each solution of FEX, used for validation studies. Triplicate 20-μl injections were made for each concentration and were chromatographed under the chromatographic conditions mentioned above. Peak area ratios were plotted against the corresponding concentrations to obtain the calibration graph for each compound.

### Forced Degradation of fexofenadine

In order to establish whether the proposed method was stability-indicating, FEX was stressed under various conditions to conduct forced degradation studies [22-24]. Peak purity test performed by photodiode array detector was useful to investigate peak purity.

All degradation experiments were performed using 1 ml of stock FEX solution (50 mg%). After exposing the drug to the studied degradation conditions, suitable dilution with the mobile phase was made to get drug concentration of 50 μg/ml.

For acid and base-induced degradation, FEX sample was heated with 2 ml of 0.5 N HCl or 0.5 N NaOH for acid and base-induced degradation, respectively, at 80°C for 4 hr and then neutralized to pH 7.0.

For oxidative degradation, FEX sample was separately heated at 80°C for 2 hr using 1 ml of each of 3% and 30% H_2_O_2_.

For photo degradations, two separate solutions of pure FEX were used. One solution was exposed to ultraviolet light (254 nm) for 8 hr, and the other was subjected to direct daylight for up to one week.

Thermal degradation was also tested by placing the drug powder in a thermostated oven at 80°C for 8 hr.

### Tablet preparation

Ten Telfast^® ^coated tablets were weighed and powdered. An accurate weight of the powder equivalent to 50.0 mg of FEX was transferred into a 100 ml volumetric flask and extracted with 60 ml methanol in an ultrasonic bath for 30 min. The solutions thus prepared were diluted to volume with the same solvent then filtered. Suitable dilutions with the mobile phase were made to prepare tablet solutions containing 30 μg/ml of FEX. Lisinopril (LIS) was added to the prepared solution so that its final concentration is 30 μg/ml. Solutions thus prepared were filtered using 0.45-mm filters (Millipore, Milford, MA, USA) then analyzed as mentioned under the construction of calibration graphs.

## Results and discussion

### Method development

In the current study, Hypersil BDS C18 column was evaluated for the purpose of separation among the active drug FEX and its four related impurities. A wavelength of 215 nm was selected for the simultaneous determination of FEX and its four impurities with high sensitivity.

To optimize the LC assay conditions, the effects of methanol percentage as well as the pH of the aqueous phase, the inclusion of ion pairing reagent (1-octane sulphonic acid) and addition of triethylamine (TEA) to the mobile phase were studied. The amphoteric nature of FEX, due to the presence of alicyclic amine and carboxylic acid moieties, contributes to the dramatic responses of the drug peaks to moderate variations in chromatographic conditions [13].

1-Octane sulphonic acid was added to the mobile phase to improve the sharpness and symmetry of FEX peaks and its related compounds. Its effect can be explained in that it acts as anionic ion pairing reagent for bases. It results in the formation of a neutral ion pair between the reagent and basic analytes. This species then undergoes mass transfer with the stationery phase and ideally leads to the separation of the components. A concentration of 0.1 gm% of 1-octane sulphonic acid in the aqueous phase was found optimum and produced maximum sharpness and symmetry of these peaks.

TEA not only provided the desired pH together with orthophosphoric acid, but also prevented peak tailing of the basic analyte, FEX, due to its silanol masking feature [22] thus permitting the separation of FEX from its related impurity B. TEA in acid medium can be used to block residual silanol groups on the silica gel backbone of bonded phase columns. This is useful for the analysis of basic ionized compounds which might interact with these silanols. In this respect, TEA is used to upset this undesirable interaction. A concentration of 1% (*v/v*) TEA in the mobile phase was found optimum in increasing the sharpness and decreasing the tailing of the measured peaks.

The percentage of the organic modifier in the mobile phase had a significant effect on the retention behavior of the studied compounds. A satisfactory separation of FEX and its four related impurities with satisfactory resolution and increased speed was obtained with a mobile phase containing 40% methanol. At lower methanol concentrations, separations occurred but with excessive tailing and increased retention times. Increasing methanol concentration led to loss of resolution and overlapping peaks.

For further optimization, 0.05 M phosphate buffers (containing 0.1 gm%-octane sulphonic acid and 1% (*v/v*) TEA) at different pH values (ranging from 2.5 to 8) were tried as the aqueous phase along with 40% methanol in the mobile phase. Interestingly very good separation was achieved on a C18 stationary phase with the mobile phase in which the pH of the aqueous phase was adjusted to 2.7. Finally the mobile phase consisting of buffer (0.05 M sodium phosphate buffer containing 0.1 gm% 1-octane sulphonic acid and 1% (*v/v*) triethylamine adjusted to pH 2.7): methanol in a ratio of (60:40, *v/v*) at a flow rate of 1.5 ml/min was found appropriate allowing adequate separation of the five compounds; FEX and its four related impurities.

**Figure 2:**
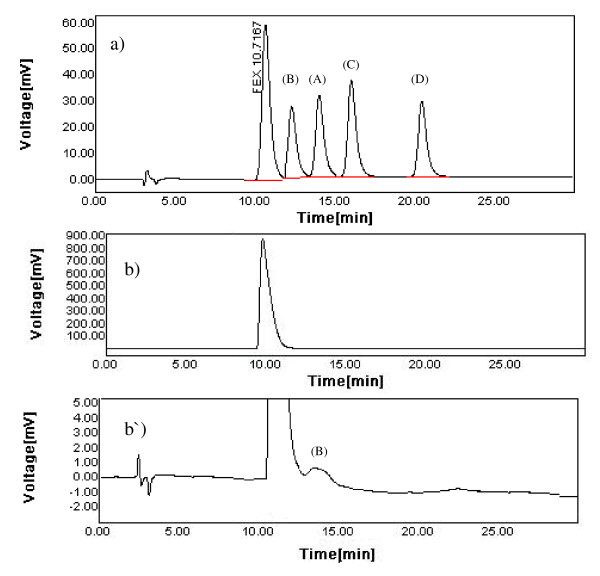
**A typical chromatogram of a standard mixture of 50 μg/ml FEX and its four related impurities; impurity A (A), impurity B (B), impurity C (C) and impurity D (D), each at a concentration of 25 μg/ml, a), a very high concentration of FEX (1000 μg/ml), b) of which the y-axis was rescaled to view the possible presence of impurities in bulk powder, b')**.

The typical chromatogram of FEX sample spiked with the four related impurities recorded using the proposed method is shown in Figure 2a. The method permitted adequate resolution of the mixture components within reasonable run-time. FEX was eluted at 10.716 min while the four related impurities were eluted at 11.987 min (impurity B), 14.013 min (impurity A), 16.530 min (impurity C) and the last eluted peak at 21.230 min being for impurity D. System suitability results of the developed method are presented in Table 1. The chromatographic characteristics of the mixture summarized indicate that the proposed HPLC method permitted adequate resolution of the mixture's components (good resolution and selectivity values) within reasonable run-time (suitable capacity factors). In addition, high column efficiency was indicated from the large number of theoretical plates. The degree of peak asymmetry was also evaluated using the tailing factor which did not exceed the critical value (1.2) indicating acceptable degree of peak asymmetry. Relative response factor (RRF) of each impurity relative to FEX peak was also calculated as mentioned in Table 1. It is the ratio of the peak response per unit concentration for each impurity to the peak response per unit concentration for the reference compound (FEX) under the given analytical conditions [18]. Since UV absorption spectra of these impurities were similar to that of FEX, their RRF were nearly equal to 1.0. In addition, low values of RSD of peak area of all studied compounds indicate good precision.

**Table 1:** Chromatographic characteristics of FEX and its four related impurities (A-D) using the proposed HPLC method

Compound	*t_R_*	*RRT*	*N*	*k*	*α*	*R_s_*	*T_f_*	RRF	*RSD* *(area precision)*
FEX	10.72	-	4940	6.2			1.000	-	0.40
					1.15	2.3			
Impurity A	11.99	1.12	5900	7.10			1.033	1.04	0.54
					1.19	3.52			
Impurity B	14.01	1.31	6210	8.47			1.123	0.91	0.65
					1.20	4.39			
Impurity C	16.53	1.81	8398	10.17			1.009	0.90	0.52
					1.31	7.54			
Impurity D	21.23	2.00	9500	13.34			1.189	0.91	0.66

*t*_*R*_: Retention time in min.

RRT: Relative retention time, of each impurity relative to FEX peak.

*K*: Capacity factor. *α*: Selectivity, between each two successive peaks.

*R_s _*:Resolution, between each two successive peaks. *T*_*f *_:Tailing factor.

RRF: relative response factor of each impurity relative to FEX peak.

RSD: relative standard deviation for area of five injections (instrument precision)

For the detection of the presence of possible impurities in bulk powder, a very high concentration of FEX (1000 μg/ml) was injected. The impurities can be identified by matching the UV spectrum and the retention times with that of standards in addition to the spiking technique. A typical chromatogram of this high concentration of FEX is shown in Figure 2b, b' and as can be seen only impurity B was detected.

### Method validation

The proposed HPLC method was validated in compliance with ICH guidelines [23-25]. The following parameters were validated. Lisinopril (LIS) was used as internal standard for the purpose of quantification of FEX and its four related compounds, being eluted at 7.117 min with sharp and symmetric peak. Other compounds were tried as internal standards, e.g. atenolol, losartan, carvedilol, simvastatin, atorvastatin and hydrochlorthiazide but none of them produced satisfactory elution relative to the peaks of FEX and its related compounds.

#### Linearity

Linearity was checked by preparing standard solutions at five different concentration levels of each of FEX and its four related compounds ranging from 0.1-50 μg/ml using a fixed concentration of 30 μg/ml LIS as internal standard. The equation for the calibration curve of FEX was *y *= 0.0527 *x*-0.025 (r = 0.9996, S_*y*/*x *_= 0.0459, S_b _= 0.0010 and S_a _= 0.0219), *y *= 0.0499 *x*+0.063, r = 0.9993 (impurity B), *y *= 0.0553 *x*+0.078, r = 0.9992 (impurity A), *y *= 0.0545 *x*-0.047, r = 0.9991 (impurity C) and *y *= 0.0498 *x*-0.084, r = 0.9995 (impurity D). High values of correlation coefficients indicating good linearity.

#### Limit of detection and limit of quantitation

Limit of detection (LOD) is defined as the concentration which has a signal-to-noise ratio of 3:1. For limit of quantitation (LOQ), the ratio considered was 10:1 with a RSD value less than 10% [26,27]. Using the proposed HPLC method, LOD and LOQ for FEX and its related impurities were calculated and were found to be 0.02 and 0.05 μg/ml, respectively.

#### Accuracy

The accuracy of the method for assay determination was checked at three concentration levels of FEX; 0.1, 10.0, and 50 μg/ml (*n *= 3) for 3 consecutive days. Solutions for the standard curves were prepared fresh every day. The percentage recoveries are tabulated in Table 2. Standard addition and recovery experiments were also conducted to determine the accuracy of the present method for the quantification of related compounds A, B, C and D. The range of addition levels of impurities to the parent compound was done at 0.15-0.75% of the concentration (10 μg/ml) of the FEX [18]. The recovery of each impurity was calculated from the slope and intercept of the calibration curve of each impurity. The mean recovery of the four impurities were found to be in the range of 98.4-102% (Table 3) indicating high degree of accuracy of the developed method.

**Table 2:** Precision and accuracy in the assay determination of FEX using the proposed LC method

Day of analysis	Spiked concentration (μg/ml)	**Mean recovery (%) ± RSD**^**a**^
Repeatability (Intra-day precision)		
Day 1	0.1	101.5 ± 0.89
	10	99.4 ± 0.49
	50	101.2 ± 0.67
Day 2	0.1	103.8 ± 0.99
	10	100.2 ± 0.67
	50	101.8 ± 0.89
Day 3	0.1	102.4 ± 0.1.2
	10	99.4 ± 0.59
	50	100.7 ± 0.55
Intermediate precision (Inter-day precision)		
	0.1	102.5 ± 1.13
	10	99.6 ± 0.46
	50	101.23 ± 0.54

^a ^Mean recovery (%) ± RSD of three determinations.

**Table 3:** Precision and accuracy of FEX related compounds using the proposed HPLC method

	Impurity A	Impurity B	Impurity C	Impurity D
Overall mean % recovery ^a^	101.5	102.0	101.9	98.4
Intra-day precision (RSD)	1.55	1.23	0.88	1.32
Inter-day precision (RSD)	1.78	1.56	1.20	1.67

^a^Overall mean % recovery of three different concentration level(1.5, 5.0, 7.5 μg/ml), (n = 9)

#### Precision

System repeatability was determined by replicate applications and measurements of peak area for FEX. Method repeatability was obtained from RSD % values obtained by repeating the assay three times on the same day (intra-day precision). Intermediate precision was assessed by the assay of sample sets on three different days (inter-day precision). The intra-and inter-day precision studies for the determination of FEX was carried out at three different concentration levels of 0.1, 10.0, 50 μg/ml (n = 3). However, precision studies for the related impurities were carried out at concentration levels of 0.15, 5.0 and 7.5 μg/ml. The calculated values of RSD% for FEX and its related impurities were calculated and mentioned in Tables 2 and 3, respectively. The results indicated high degree of repeatability and intermediate precision of the proposed method.

#### Robustness

To evaluate the HPLC method robustness, a few parameters were deliberately varied. The parameters included slight variation in methanol percentage in the mobile phase (38, 40, 42), pH of the aqueous phase (2.5, 2.7, 2.9), flow rate (1.3, 1.5, 1.7 ml/min), injection volume (19.5, 20, 20.5 μl), wavelength of detection (213, 215, 217), column temperature (23, 25, 27°C) and methanol of different lots. Robustness of the method was done at the same concentration levels as those used for the evaluation of the precision. Insignificant differences in peak areas (RSD < 2%) and slight variability in *k *values (RSD < 1.8%) were observed for FEX and related impurities.

#### Specificity

Specificity is the ability of the method to accurately measure the analyte response in the presence of all potential sample components.

To demonstrate the specificity of the method, the impurities discussed above (A-D) were added to pure FEX sample and the mixture was analyzed for assay and the results were compared with pure sample results. Reproducibility was observed in both the cases (RSD < 2.0). The specificity of the HPLC method was also assessed by the complete separation of FEX in presence of its related impurities along with other parameters like retention time (*t_R_*), capacity factor (*k*), tailing factor (*T_f_*), etc. (Table 1). The peaks obtained were sharp and had clear baseline separation (Figure 2a).

#### Forced degradations

Accelerated degradation studies were carried out in order to provide an evidence for the specificity of the proposed method.

The chromatograms of the samples treated with acid, base, hydrogen peroxide, photochemical and dry heat, showed well separated peaks of pure FEX. Summary of all degradation studies was mentioned in Table 4.

**Table 4:** Summary of degradation studies of FEX using the proposed HPLC method*

	% Recovery	Purity angle	Purity threshold	Match angle	Match threshold	***t***_***R ***_**valuesof degradation products**
Acid-induced degradation (0.5 N HCl, 80°C, 4 hr)	82.51	0.130	0.347	0.069	1.079	17.48, 19.62, 26.40
Base-induced degradation (0.5 N NaOH, 80°C, 4 hr)	89.54	0.145	0.425	0.081	1.105	-
Oxidative degradation	89.73	0.125	0.332	0.084	1.071	-
-3% H_2_O_2_						
(80°C, 2 hr)						
-30% H_2_O_2_ (80 s°C, 2 hr)	22.01	0.145	0.352	0.035	1.077	11.83
Photochemical degradation	98.89	0.121	0.341	0.111	1.076	-
-Direct daylight						
(7 days)						
-UV at 254 nm (8 hr)	98.66	0.111	0.342	1.076	1.098	-
Thermal degradation (80°C, 8 hr)	98.40	0.118	0.411	0.101	1.098	-

*FEX peak was defined as main peak since match angle was less than match threshold and also as pure peak since purity angle was less than purity threshold under all forced tests.

#### Acid- and base-induced degradation

**Figure 3:**
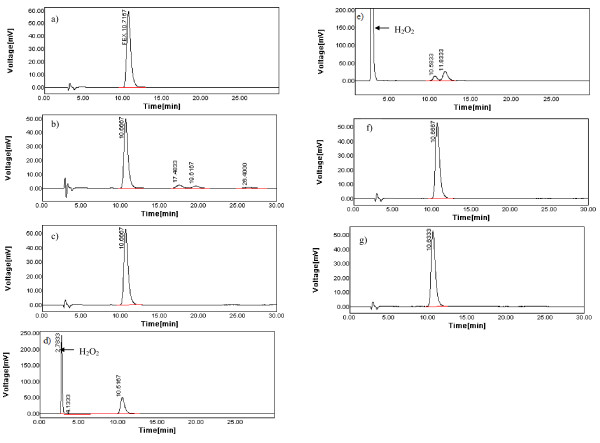
**A typical chromatogram of a standard solution containing 50 μg/ml FEX, a) and and its corresponding acid degradation, b), base-degradation, c), oxidative-degradation with 3% H_2_O_2_, d), oxidative-degradation with 30% H_2_O_2_, e), day light degradation, f) and thermal degradation, g)**.

FEX peak detected at about 10.7 min showed drug recovery at the level of 82.51% and 89.54% from the acid and base stressed samples, respectively. For acid-induced degradation, three secondary degradation peaks were detected at 17.48, 19.62 and 26.40 min. However, for base-induced degradation, no degradation peaks were recorded under the studied conditions (Figures 3b, c).

#### Hydrogen peroxide-induced degradation

The chromatogram of the FEX sample treated with 3 and 30% (*v/v*) H_2_O_2 _showed a significant degradation of FEX (% recovery were 89.73 and 22.01, respectively). A secondary degradation peak was detected at 11.83 min with only 30% H_2_O_2_. The peak of H_2_O_2 _did not interfere with the analysis being eluted at 2.78 min (Figures 3d, e).

#### Photo degradation

Photodegradation of FEX was studied using direct daylight (up to 1 week) and UV light (254 nm). No significant degradation of FEX was reported after the exposure of drug solutions to direct daylight for up to one week or to UV light (254 nm) for up to 8 hrs (Figure 3f).

#### Thermal degradation

Heating the drug powder in a thermostated oven at 80°C for 8 hr produced nearly no effect on FEX peak indicating its stability to thermal degradation under the studied conditions (Figure 3g).

The number of degradation products with their *t*_R _values and % recovery of FEX were calculated and are given in Table 4. The chromatographic peak purity tool was applied to verify FEX peaks, showing 100% purity in all cases. This was performed by calculating purity angle and purity threshold for FEX peaks. In all cases, FEX peak was defined as main peak since match angle was less than match threshold and also as a pure peak since purity angle was less than purity threshold under all forced tests. This showed that FEX peak had no detectable impurity peaks embedded in and are free of co-eluting degradation compounds. Besides, it was observed that peaks of FEX present appropriate resolution and base-line separation and they were not affected by degradation. From the above results, it is clear that the proposed method can be used as a stability indicating method for determining the stability of FEX in bulk and pharmaceutical formulations.

#### Excipients interference

**Figure 4:**
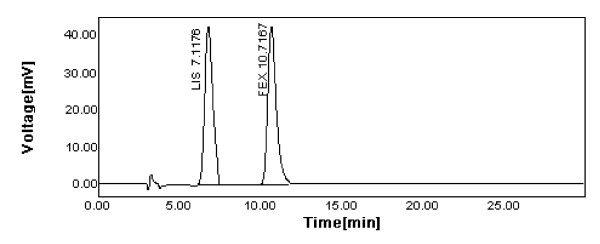
**A chromatogram of the prepared tablet solution containing 30 μg/ml of each of FEX and the internal standard; LIS**.

The excipients present in pharmaceutical tablets of FEX did not show any interference with FEX peak since no excipients peaks appear in the chromatogram of the prepared tablet (Figure 4).

#### Analysis stability

##### Stability of assay solutions

Standard and sample solutions containing 50.0 μg/ml of FEX were prepared and stored at room temperature, protected from light, for 20 hr. They were then injected into the LC system. Since no additional peaks were found in the chromatogram with no reduction in the peak area, this indicates stability of both standard and sample solutions for about 20 hr.

##### Mobile phase stability

Moreover, mobile phase stability was also checked. This was performed by eluting standard and sample solutions containing 50.0 μg/ml of FEX using fresh and aged mobile phase (left for 7 days). It was concluded that mobile phase preparation was found to be stable for about 7 days since aged mobile phase produced equivalent chromatography and results to that obtained with fresh mobile phase.

### Application to commercial tablets

Using the proposed HPLC chromatographic method, assay of FEX in tablets was carried out as described under the experimental section. Five replicate determinations were made. Satisfactory results were obtained and were in a good agreement with the label claim (Table 5). The results of determination of FEX in tablets obtained from the suggested HPLC method were compared with those of a reference LC method [16]. Moreover, to check the validity of the proposed methods, the standard addition method was applied by adding FEX to the previously analyzed tablets. The results of analysis of the commercial tablets and the recovery study (standard addition method) of FEX (Table 5) suggested that there is no interference from any excipients, which are normally present in tablets. Statistical comparison of the results was performed with regard to accuracy and precision using Student's t-test and the variance ratio F-tests at 95% confidence level (Table 5). Since the calculated t- and F-values did not exceed the theoretical ones, this indicated that there was no significant difference between the two methods of analysis [26,27]. A typical HPLC chromatogram shown in Figure 4 indicates that FEX and the internal standard are well separated in the formulation sample and no impurities were detected in the analyzed concentration level.

**Table 5:** Determination of FEX in commercial tablets* by the proposed LC method

	**Mean found ± RSD%**^**a**^	
	
	Proposed LC method	**Reference method **[16]
	98.97 ± 0.49	99.22 ± 1.07
	F^b ^= 4.768	
	*t*^b ^= 0.992	
Recovery^c^		
	99.15 ± 0.65	99.33 ± 0.24

*Labelled to contain 120 mg FEX per tablet, batch number 0T00757.

^a ^Mean and RSD% for five determinations

^b ^Theoretical values of F and *t *for p = 0.05 and n = 5 are 6.39 and 2.31, respectively.

^c ^For standard addition of 50% at the nominal content (n = 5)

## Conclusion

A simple, specific and accurate RP-LC method with a photodiode array detector has been developed and validated for the analysis of fexofenadine HCl along with its four related compounds; keto fexofenadine (Impurity A), meta isomer of fexofenadine (Impurity B), methyl ester of fexofenadine (Impurity C) in addition to the methyl ester of ketofexofenadine (Impurity D). The method is very economical, because it required neither the use of a chiral stationary phase nor addition of chiral additives to the mobile phase; the inexpensive phosphate buffer, 1-octane sulphonic acid, triethylamine in addition to methanol as the organic modifier were used as the mobile phase along with with a C18 RP-LC column; these are available in every chromatography laboratory. The developed method was fully validated as per ICH guidelines [23-25]. The method provides simple, accurate, precise and stability-indicating assay for the determination of FEX along with its four related compounds in bulk powder and pharmaceutical tablets, without interference from the excipients and in the presence of acidic, alkaline, oxidative, thermal and photolytic degradation products. All of the degradation products were well separated from the drug substances demonstrating the stability-indicating power of the method. Thus the developed method is a stability-indicating assay that can be widely used for the routine analysis of FEX and its related impurities, in bulk powder and pharmaceutical tablets without any interference.

## Abbreviations

HPLC: high-performance liquid chromatography; DAD: diode array detector; BP: british pharmacopoeia; USP: united states pharmacopoeia; FEX: fexofenadine; Impurity A: keto fexofenadine; Impurity B: meta isomer of fexofenadine; Impurity C: methyl ester of fexofenadine; Impurity D: methyl ester of keto fexofenadine; LIS: lisinopril; TEA: triethyl amine; ICH: International Conference on Harmonization; UV: ultraviolet; LOD: limit of detection; LOQ: limit of quantification; SD: standard deviation; RSD: relative standard deviation; (*t_R_*): retention time; (*k*): capacity factor; (*T_f_*): tailing factor.

## Competing interests

The authors declare that they have no competing interests.

## Authors' contributions

HMM supervised the practical work, analyzed the data statistically and participated in writing the manuscript. MAS proposed, planned and supervised the whole work. IVO carried out the experimental work. All authors read and approved the final manuscript.
